# Investigation of COVID-19 outbreak at a refugee transit centre, Kisoro District, Uganda, June–July 2022

**DOI:** 10.1371/journal.pgph.0002428

**Published:** 2024-03-06

**Authors:** Peter Kawungezi, Richard Migisha, Robert Zavuga, Brenda Nakafeero Simbwa, Jane Frances Zalwango, Mackline Ninsiima, Thomas Kiggundu, Brian Agaba, Irene Kyamwine, Daniel Kadobera, Benon Kwesiga, Lilian Bulage, Robert Kaos Majwala, Alex Riolexus Ario

**Affiliations:** 1 Uganda National Institute of Public Health, Uganda Public Health Fellowship Program, Kampala, Uganda; 2 Ministry of Health, Kampala, Uganda; 3 Department of Global Health Security, Baylor Uganda, Kampala, Uganda; Fundacao Oswaldo Cruz, BRAZIL

## Abstract

Due to conflict in the Democratic Republic of Congo (DRC), approximately 34,000 persons arrived at Nyakabande Transit Centre (NTC) between March and June 2022. On June 12, 2022, Kisoro District reported >330 cases of COVID-19 among NTC residents. We investigated the outbreak to assess its magnitude, identify risk factors, and recommend control measures. We defined a confirmed case as a positive SARS-CoV-2 antigen test in an NTC resident during March 1–June 30, 2022. We generated a line list through medical record reviews and interviews with residents and health workers. We assessed the setting to understand possible infection mechanisms. In a case-control study, we compared exposures between cases (persons staying ≥5 days at NTC between June 26 and July 16, 2022, with a negative COVID-19 test at NTC entry and a positive test at exit) and unmatched controls (persons with a negative COVID-19 test at both entry and exit who stayed ≥5 days at NTC during the same period). We used multivariable logistic regression to identify factors associated with contracting COVID-19. Among 380 case-persons, 206 (54.2%) were male, with a mean age of 19.3 years (SD = 12.6); none died. The attack rate was higher among exiting persons (3.8%) than entering persons (0.6%) (p<0.01). Among 42 cases and 127 controls, close contact with symptomatic persons (aOR = 9.6; 95%CI = 3.1–30) increased the odds of infection; using a facemask (aOR = 0.06; 95% CI = 0.02–0.17) was protective. We observed overcrowding in shelters, poor ventilation, and most refugees not wearing face masks. The COVID-19 outbreak at NTC was facilitated by overcrowding and suboptimal use of facemasks. Enforcing facemask use and expanding shelter space could reduce the risk of future outbreaks. The collaborative efforts resulted in successful health sensitization and expanding the distribution of facemasks and shelter space. Promoting facemask use through refugee-led efforts is a viable strategy.

## Introduction

Coronavirus Disease 2019 (COVID-19), is a disease caused by severe acute respiratory syndrome coronavirus 2 (SARS-CoV-2) [1]. Refugee settings are especially susceptible to outbreaks of infectious diseases, including COVID-19 [2,3]. The susceptibility is linked to overcrowding, inadequate access to clean water and soap, and constraints on hand-washing facilities [3–5]. All these challenges, coupled with immediate concerns of safety, sustenance, and shelter, significantly limit infection prevention and control (IPC) efforts in these settings [6–9]. In addition, early detection of the virus is often not possible because monitoring access to the camp is difficult, and thus not easy to know whether people infected are camp residents or people from outside the camp [7].

COVID-19 outbreaks have been reported among refugees in Bangladesh [7,8], Greece[7,10,11], and Brazil [12]. The COVID-19 preventive measures among refugees are similar to those in the general population, including mass testing, vaccination, social distancing, use of face masks, hand washing, and other measures to improve personal hygiene [4,8,13–15]. However, the implementation of these measures among refugees may be limited [4,11,12]. Innovative approaches, including refugee-led initiatives, have demonstrated successful contributions to addressing specific needs of refugee communities, with refugees taking on roles as providers of protection and assistance [16–18]. These efforts could go beyond offering leadership, conflict resolution, life skills, to support IPC measures.

In March 2022, a new influx of more than 10,000 refugees fled to Uganda’s southwestern Kisoro District due to violent clashes in DRC. Refugees entered through the Bunagana border and were relocated to Nyakabande Transit Centre (NTC) [19]. This was during an ongoing COVID-19 outbreak in Uganda that registered a total of 163,994 COVID-19 cases by April, 2022, with the omicron variant being the predominant variant[20]. On 4^th^ April 2022, the NTC registered the first case of COVID-19 through mandatory screening at entry and exit that had been implemented since 28^th^ March 2022. Cumulative COVID-19 cases at NTC increased to 621 by June 30, 2022 [20]. By August 30, 2022, together with the Bubukwanga Transit Center in Bundibugyo District, the two registered a total of 1,365 COVID-19 cases [21].

We investigated the outbreak to establish its scope, identify factors associated with COVID-19 infection in NTC, and to recommend control and preventive measures for the future.

## Methods

### Ethics statement

This outbreak investigation was in response to a public health emergency and was therefore determined to be non-research. The Ministry of Health gave a directive to investigate this outbreak and the office of the Center for Global Health, US Center for Disease Control and Prevention determined that this activity was not human subject research and that its primary intent was for public health practice or disease control. The case control study being part of the outbreak investigation, was also exempted from ethical approval by the Ministry of Health. The authors sought permission to conduct the investigation from District Health authorities of Kisoro District. Permission was also sought from the administrators of the Nyakabande Transit Centre to access data about refugees. The authors sought verbal informed consent from the respondents who were COVID-19 case patients, and key informants. They were all informed that their participation was voluntary and their refusal would not attract any negative consequences. Authors did not have access to information that could identify individual participants during or after the data collection since the data collected did not contain individual personal identifiers. The verbal consent was by the interviewer before the interview and was documented on every record as Yes/No. the verbal consent was approved by the Ministry of Health and the office of the Center for Global Health, US CDC.

### Outbreak area

**Fig 1:**
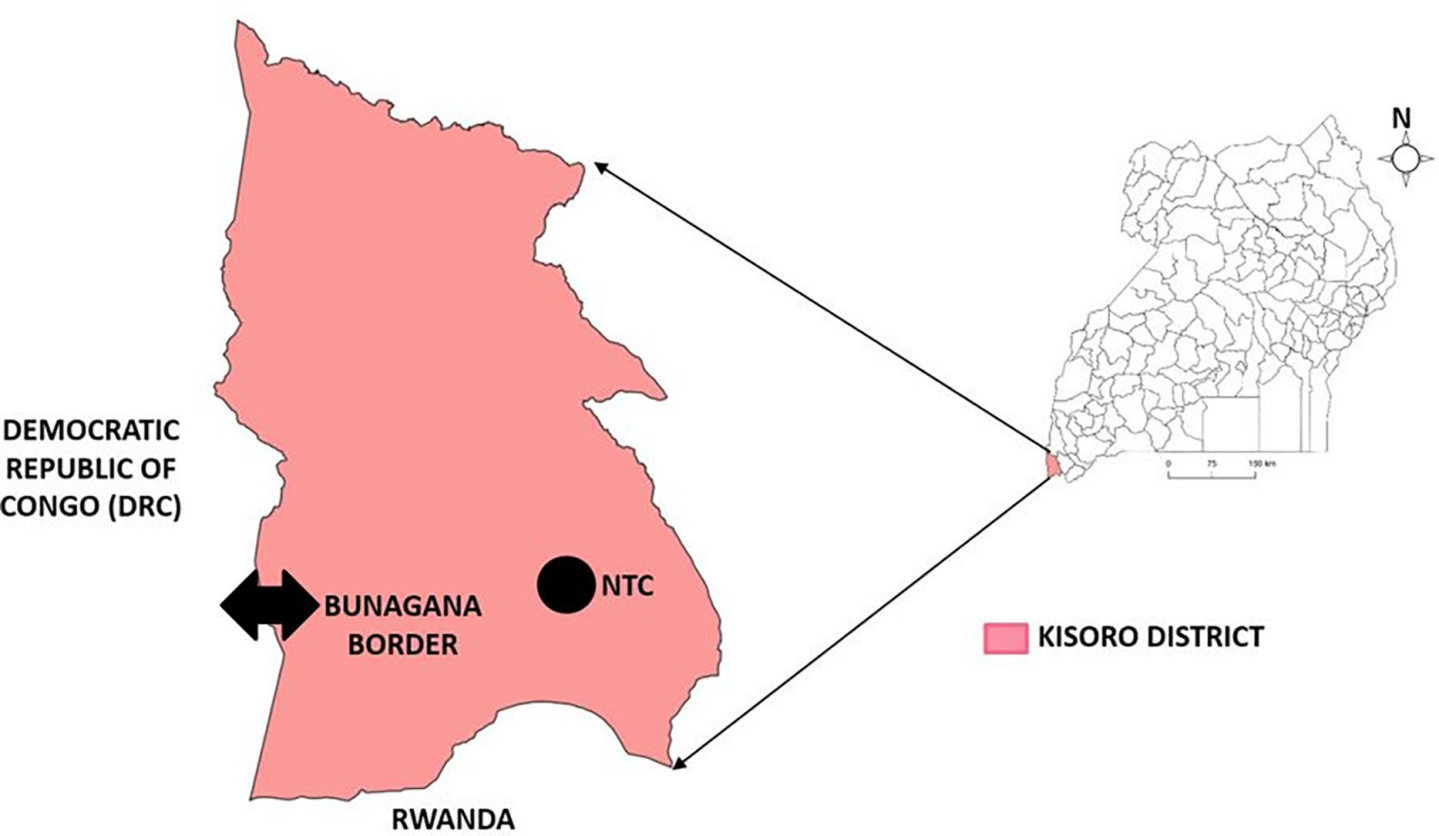
Location of Nyakabande Transit Centre (NTC), Kisoro District, Southwestern Uganda (Map drawn using QGIS 3.22, link to base layer shape file. https://www.naturalearthdata.com/downloads/).

NTC is located 5km from Kisoro Town and 18 Kilometers from Bunagana Border in Kisoro District, South West Uganda. Kisoro District shares boundary with Democratic Republic of Congo (DRC) in the west and the Republic of Rwanda in the south (Fig 1). It was opened in 1994 to cater for both returning and arriving refugees fleeing the genocide in Rwanda. In 2022, the capacity of NTC was 825 individuals. Refugees are supposed to stay for a period of 2–5 days in the transit centre then be transferred to settlement camps. However, due to the 2022 influx, this was not the case as some refugees stayed longer in the transit centre in anticipation to return to their home country.

All refugees were mandated to be tested for COVID-19 at entry to NTC, and at the time of exit to the resettlement camp. All refugees that tested positive both at entry and exit were subsequently treated in the COVID-19 isolation unit. Refugees were also mandated to be vaccinated against COVID-19 at the time of relocation to the resettlement camp if they were eligible. In early April 2022, NTC management designated one shelter for isolation of COVID-19 confirmed case patients.

### Case definition and case finding

We defined a confirmed case as a positive SARS-CoV-2 antigen test (SD Biosensor, Inc., Republic of Korea) in a resident of NTC during March 1–June 30, 2022. We abstracted data from the COVID19 laboratory testing and isolation registers using a data abstraction tool to identify case-patients for the COVID-19 tests at entry and exit of NTC. Between March 28, 2022 and June 26, 2022, data was collected on the total number of refugees tested for COVID-19 at entry and exit, as well as the total number of positive test results at both entry and exit to determine when refugees were testing positive in relation to their stay at the transit centre and to identify whether the COVID-19 cases were imported or contracted while at the centre.

### Descriptive epidemiology

We calculated mean age, and proportions by sex of the case-patients. We were not able to calculate attack rates by age or sex because there were no data on population by age and sex. We generated an epidemiological curve for the distribution of COVID-19 positive tests by date of testing among refugees in NTC between April to June 2022 and stratified the curve by the time of testing whether at time of relocation or at time of entry and the distribution of COVID-19 cases by symptom onset date among refugees in NTC between June to July 2022. We calculated the COVID-19 test positivity rate for tests done at entry and exit as the number of positive tests divided by number of total tests done.

### Environmental assessment

We assessed the infrastructure and IPC of the NTC premises and the COVID-19 isolation unit. We observed sanitation and hygiene practices, availability of water, hand washing facilities, use of facemasks, crowding, the structure and status of shelters within the NTC. We did not consider the health, nutritional or mental health status of refugees.

### Hypothesis generation

We conducted four hypothesis-generating key informant interviews with the District Health Officer, Chairperson Kisoro District Local Government, District Surveillance Focal person, NTC Camp commandant, and Medical Teams International (MTI)’s clinical lead officer. We also conducted semi structured interviews with nineteen case-patients in the COVID-19 isolation unit. We explored factors related to non-adherence to preventive measures.

### Unmatched case control study

We conducted an unmatched case control study with refugees after verbal informed consent. We defined a case as a person who had a negative COVID-19 test at entry and positive COVID-19 test at exit and had stayed ≥5 days [22] at NTC between June 26 and July 16, 2022. A control was defined as person who had a negative COVID-19 test at both entry and exit and had stayed ≥5 days at NTC between June 26 and July 16, 2022. The outcome variable was COVID-19 positive at exit (for a case) and COVID-19 negative test at exit (for a control) given a negative COVID-19 test at entry. For each case and control, we collected data on age, sex, education level, moving out of the centre, frequency of moving out (short trips to the shop, bars or trading centre) of the centre, interaction with host community, having COVID-19 symptoms at time of COVID -19 test at exit, cigarette smoking, underlying medical illnesses, use of facemasks, handwashing behavior, COVID-19 vaccination before entry to the center, date of last COVID-19 vaccination before entry to the center. The case-patients were identified at the COVID-19 isolation unit while the controls were identified within the camp with help of the laboratory register before being transferred to resettlement areas.

We collected data using an electronic tool in Kobocollect and exported to Epi info version 7.2.5.0 for analysis. We used logistic regression to identify factors associated with COVID-19 infection. Variables that had a p-value <0.2 at bivariate analysis were included in the final model for multivariate analysis in a backward stepwise approach. Corresponding adjusted odds ratios (aORs) and 95% confidence intervals were reported. The final level of significance was considered at a p-value <0.05 [23].

## Results

### Descriptive epidemiology

**Fig 2:**
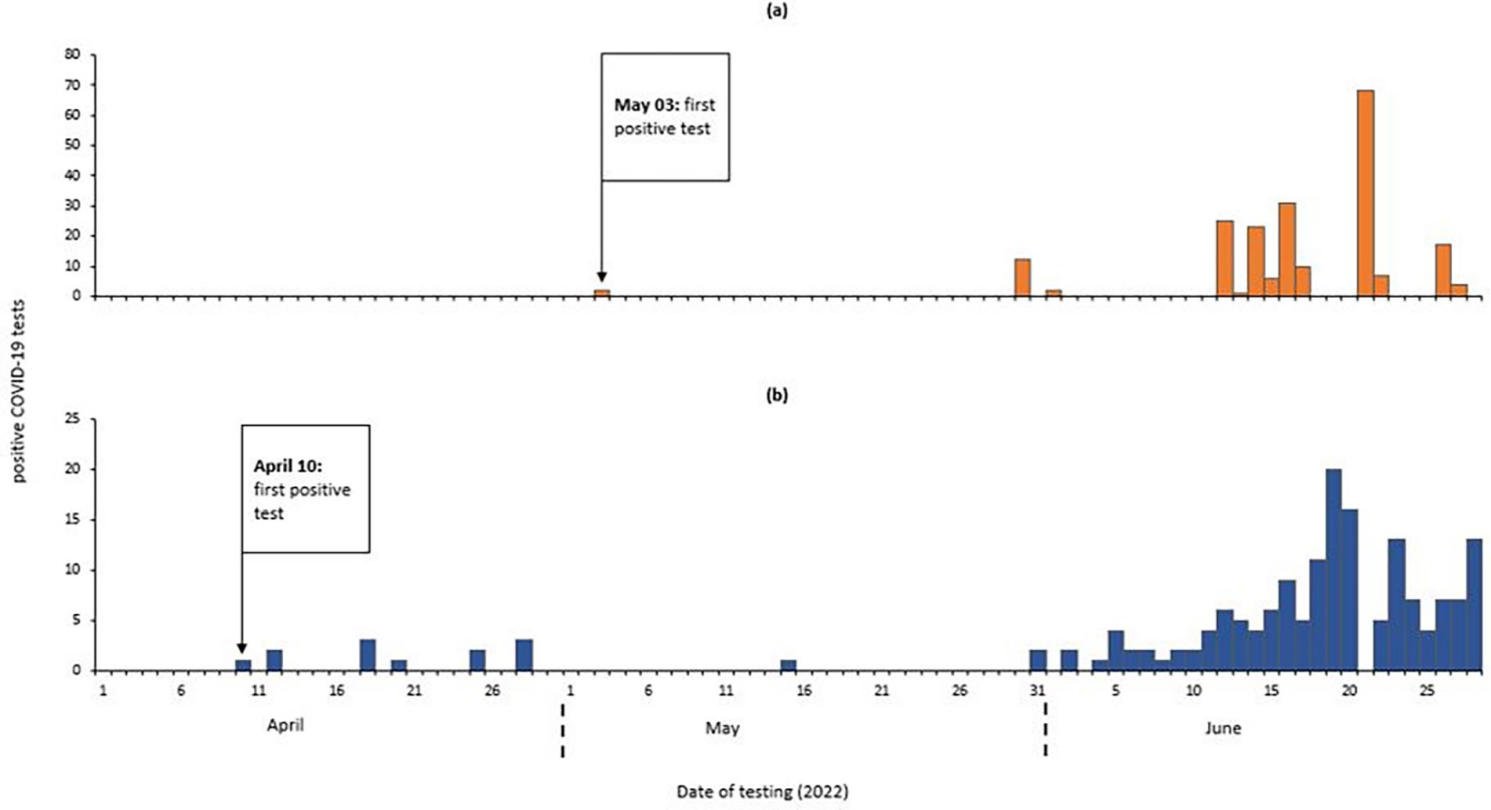
Distribution of COVID-19 positive tests by date of testing among refugees in Nyakabande Transit Centre, Kisoro District, Uganda, April-June 2022(a)-tests done at time of relocation (b)-tests done at time of entry.

We identified 380 confirmed case-persons and no deaths. Of these 206 (54.2%) were male. The mean age was 19.3 years (SD±12.6). The first positive test among new arrivals was registered on April 10, 2022 and among individuals relocating to resettlement camps on May 3, 2022 (Fig 2).

Out of 34,690 tests at entry, 209 (0.6%) tested positive. Out of 8,260 tests done at exit, 318 (3.8%) tested positive.

**Fig 3:**
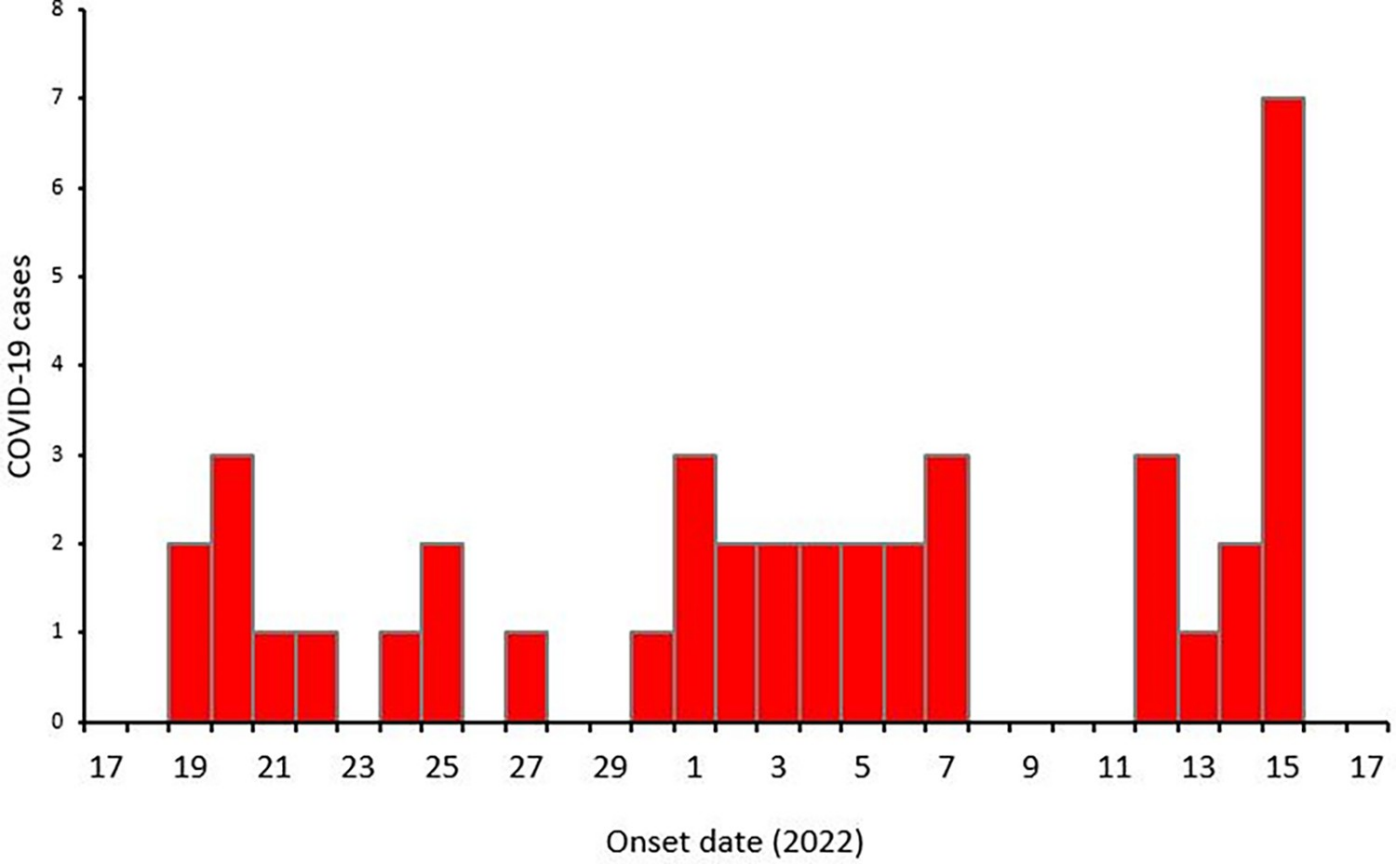
Distribution of COVID-19 cases by symptom onset date among refugees in Nyakabande Transit Centre, Kisoro District, Uganda, June–July 2022.

The epidemic curve for identified COVID-19 cases had three successive peaks (Fig 3).

### Environmental assessment findings

We observed crowding among refugees, especially at the screening area on arrival, within the shelters, and during the lining up for food and relief items. This was similar to what was reported in the key informant interviews that revealed that shelters were at twice their capacity and the isolation unit was at three times its capacity. Upon entry, each refugee was given one cloth face mask. However, there was limited adherence to mask-wearing guidelines among refugees in the transit center. Furthermore, among those observed wearing masks, many were using them incorrectly by wearing them only over their chin rather than covering both their nose and mouth. While hand washing facilities with running water were readily available, there was a noted deficiency in observed hand washing practices among refugees.

### Hypothesis generation interview findings

#### Key informant interviews

Key informant interviews revealed that the number of refugees were beyond the holding capacity of the NTC, most of refugees did not use face masks despite each being given a mask at entry, and that refugees had other bothering concerns like safety, family, food, among others than the concern of contracting COVID-19 as highlighted in the following quotes………….*“This is overwhelming… as we speak now… the shelters have 1*,*600 individuals yet they have a total capacity of eight hundred twenty-five…the isolation unit that we set up for twenty-five patients… sometimes can have sixty-five patients…”* **Nyakabande transit centre leader**

Refugees were reported not use face masks even after providing them with free face masks.

#### “*we give every refugee one cloth facemask… but most of them do not use their facemasks…*” Nyakabande transit centre clinical lead

It was also reported that most refugees were concerned about the safety of their lives, property, family and what to eat including the concern about their conjugal rights. It was thought that they could have been prioritizing these over the COVID-19 control measures.

*“When refugees come here to the transit centre… they still think they are not safe… they think of danger any time… worry about their property and family… what to eat… I have heard many complaining that they can’t have their conjugal rights…”* **Kisoro District political leader**

#### Structured interviews with the COVID-19 patients at the isolation units

Out of the 19 case-patients interviewed, 6(32%) did not wear face masks at all, 3(16%) reported close interaction with a symptomatic person before developing the illness.

Based on the descriptive epidemiology and the hypothesis generation interview findings, we hypothesized that crowding and non-compliance to personal protective measures was associated with an increased risk of contracting COVID-19 at NTC.

### Case control findings

In a case control study, we enrolled 42 cases and 127 controls. The cases were comparable to controls across age, sex, and level of education. Thirty-three (78.6%) of the cases were aged between 5–29 years. Thirty-four (89.5%) of cases had neither attended school nor completed primary education (Table 1).

**Table 1:** Socio-demographic characteristics of cases and controls in Nyakabande Transit Centre, Kisoro District, Uganda, June–July 2022.

Variable	Cases (n = 42)		Controls (n = 127)		P-value
	n	(%)	n	(%)	
**Sex**					0.56
Male	21	(50.0)	57	(44.9)	
Female	21	(50.0)	70	(55.1)	
**Age** ^**ð**^					0.69
5–29	33	(78.6)	94	(74.0)	
30–49	6	(14.3)	26	(20.5)	
≥50	3	(7.1)	7	(5.5)	
**Education level** *					0.14
None or Primary	34	(89.5)	62	(77.5)	
Secondary or Tertiary	4	(10.5)	18	(22.5)	

^**ð**^ Median age = 24 (range, 21–29) years for both cases and controls

* sample size considered was cases (n = 38) controls (n = 80).

In the multivariate analysis, using a facemask (aOR = 0.06, 95% CI 0.02–0.16) reduced the odds of infection by 94%. The odds of contracting COVID-19 infection were 10 times higher among refugees who had close contact with a COVID-19 symptomatic person compared to refugees who did not have such close contact (aOR = 10.34, 95%CI 3.29–32.55) (Table 2).

**Table 2:** Factors analyzed for association with COVID-19 infection among refugees in Nyakabande Transit Centre, Kisoro District, Southwestern Uganda, June–July 2022.

Variable	Cases (n = 42)		Controls (n = 127)		cOR	(95% CI)	aOR	(95% CI)
	n	(%)	n	(%)				
**Sex**								
Male	21	(50)	57	(44.9)	Ref.			
Female	21	(50)	70	(55.1)	1.23	(0.61-2.47)		
**Age in years**								
5–29	33	(78.6)	94	(74.0)	Ref.		Ref.	
30–49	6	(14.3)	26	(20.5)	0.66	(0.25–1.74)	0.94	(0.29–2.97)
≥50	3	(7.1)	7	(5.5)	1.22	(0.30–5.00)	3.38	(0.57–20.07)
**Education level** ^**∞**^								
None or Primary	34	(89.5)	62	(77.5)	Ref.		Ref.	
Secondary or Tertiary	4	(10.5)	18	(22.5)	2.47	(0.73–10.8)	1.07	(0.28–4.07)
**Moving out of the center**								
Moves out	28	(66.7)	101	(79.5)	Ref.		Ref.	
Does not move out	14	(33.3)	26	(20.5)	0.51	(0.23–1.12)	2.49	(0.86–7.25)
**Frequency of moving out in a week** *								
Once	11	(39.3)	27	(26.7)	Ref.			
More than once	17	(60.7)	74	(73.3)	0.56	(0.23–1.36)		
**Interact with the host community** *								
No	16	(57.1)	53	(52.5)	Ref.			
Yes	12	(42.9)	48	(47.5)	1.2	(0.52–2.81)		
**Cigarette smoking**								
No	41	(97.6)	115	(90.5)	Ref.		Ref.	
Yes	1	(2.4)	12	(9.5)	4.28	(0.59–7.28)	0.52	(0.05–4.91)
**Close contact with a COVID-19 symptomatic person**								
No	26	(61.9)	109	(85.8)	Ref.		Ref.	
Yes	16	38.1)	18	(14.2)	0.27	(0.12–0.60)	10.34	(3.29–32.55)
**Having a family member with COVID-19 in NTC seven days before the exit test**								
No	40	(95.2)	116	(91.3)	Ref.			
Yes	2	(4.8)	11	(8.7)	1.90	(0.40–8.93)		
**Using facemask**								
No	27	(64.3)	20	(15.8)	Ref.		Ref.	
Yes	15	(35.7)	106	(84.2)	9.63	(4.36–21.25)	0.06	(0.02–0.16)
**COVID-19 vaccination before entry**								
No	40	(95.2)	122	(96.1)	Ref.			
Yes	2	(4.8)	5	(3.9)	1.22	(0.23–6.53)		

cOR: Crude Odds Ratio; aOR: Adjusted Odds Ratio; CI: Confidence Interval; Ref: Reference category; NTC: Nyakabande Transit Centre

*Sample size considered were cases (n = 28) and controls (n = 101)

^**∞**^ sample size considered was cases (n = 38) and controls (n = 80).

## Discussion

The investigation showed that the COVID-19 positivity rate at the exit was six times higher than that at the entry to NTC suggesting COVID-19 transmission within the transit centre. The epidemic curve had three successive with one incubation period apart suggesting a propagated epidemic. Having close contact with a symptomatic person increased the odds of COVID-19 infection. Overcrowding and failure to use facemasks among refugees likely fueled the outbreak.

In this outbreak, being in close proximity to someone exhibiting COVID-19 symptoms raised the likelihood of contracting the virus. The congestion observed in the transit center likely facilitated the close interaction among refugees. Overcrowding has been identified as a common risk factor for COVID-19 infection among refugees in various places. For instance, a study conducted in refugee camps in Bangladesh reported a higher prevalence of COVID-19 in densely populated camps [24]. Similarly, another study in Jordan found revealed that overcrowding was a contributing factor to COVID-19 infections among Syrian refugees living in urban areas [9]. To mitigate this risk, it is crucial to expanding shelter capacity and enforce social distancing measures, particularly during the distribution of essential items like food to minimize close contact among refugees.

Based on our study results, the use of facemasks was highly effective in protection against COVID-19. Facemasks consistently reduce COVID-19 risk in various settings, including prisons [6,9,25,26]. Community-wide face mask use mandates can effectively curb transmission [27], hence the widespread use in public spaces during the COVID-19 pandemic [28–31]. However, most refugees did not wear the facemasks provided to them. This hesitancy could be attributed to the challenges that refugees routinely encountered, prioritizing immediate concerns like safety, sustenance, and shelter over preventive measures [7–9,32]. In this study, we observed refugees not using the facemasks provided to them. These findings align with findings in a study conducted in a refugee setting in Bangladesh, majority of refugees did not maintain consistent use of facemasks, even when these were made available to them [26]. Promoting the use of facemasks among refugees through refugee-led efforts could improve the protection [17,18]. A study in Jordan highlighted the significant role of face masks in reducing viral transmission among Syrian refugees in crowded urban areas, reinforcing the need for promoting their use in similar settings [9].

The investigation had limitations that should be considered when interpreting the results. Firstly, there was a challenge in following up with refugees who met the case definition but had been transferred to resettlement camps due to logistical constraints. The limitation might have led to underestimation of the study’s outcomes, as the true impact on these individuals could not be accurately assessed. Secondly, the study faced limitations related to data completeness. Specifically, there were incomplete records regarding the sex and age of refugees in the registers at the National Tuberculosis Center (NTC). This data gap could have led to an underestimation of the disease burden, as a comprehensive understanding of the demographic characteristics of the refugee population is essential for a more accurate assessment of COVID-19’s impact within this group. Lastly, the retrospective nature of the investigation could have introduced potential sources of bias. Specifically, participants may have been susceptible to recall bias, where their ability to accurately remember and report past experiences and behaviors could have been influenced. Additionally, there might have been social desirability bias as participants may have felt inclined to provide responses that they believed were socially acceptable or expected. These biases could have led to an overestimation of the effects of associated factors on COVID-19 infection within the study, as the reported data may not fully reflect the actual experiences and behaviors of the participants.

## Conclusion

In conclusion, the COVID-19 outbreak at NTC was facilitated by overcrowding and failure to use personal protective measures. Propagated epidemics and heightened infection risks with close contact emphasize the need for urgent interventions. Enforcing face mask use and expanding shelter space at NTC could reduce the risk of future outbreaks. Promoting facemask use through refugee-led efforts is a viable strategy.

### Public health actions

Following the dissemination of our findings, a refugee-led health sensitization program focused on educating refugees about the use of facemasks was organized in collaboration with the clinical lead at the NTC. The training emphasized the risks of COVID-19 infection, the correct way to wear a facemask, and the benefits of using facemasks to protect one’s own health and that of others in the community. We observed a positive change in facemask usage behavior among refugees, and those who did not have facemasks before began to request for them. We recommend that this health talk on facemask usage be continued on a daily basis.

We organized advocacy meetings with the NTC camp management and Kisoro District Local Government to discuss the urgent need for expanding shelter space and increasing the procurement and distribution of facemasks. Our goal was to ensure that every refugee had access to at least two facemasks. The leadership was responsive to our proposal and mobilized implementing partners to procure more facemasks. Furthermore, the NTC management prioritized the expansion of shelter space as an intermediate-term action to be taken.

## Supporting information

S1 Checklist STROBE statement—checklist of items that should be included in reports of observational studies. (DOCX)

S1 Text Questionnaire. (DOCX)

## References

[1] WHO WHO. Coronavirus disease (COVID-19) 2022 [Available from: https://www.who.int/health-topics/coronavirus#tab=tab_1.

[2] Orendain DJA, Djalante R. Ignored and invisible: internally displaced persons (IDPs) in the face of COVID-19 pandemic. Sustainability science. 2021;16(1):337–40. doi: 10.1007/s11625-020-00848-0 32837575 PMC7406698

[3] Lau LS, Samari G, Moresky RT, Casey SE, Kachur SP, Roberts LF, et al. COVID-19 in humanitarian settings and lessons learned from past epidemics. Nature Medicine. 2020;26(5):647–8. doi: 10.1038/s41591-020-0851-2 32269357

[4] UNHCR TURA-A. 2021. [cited 2022]. Available from: https://www.unhcr.org/health-covid-19.html.

[5] Hargreaves S, Kumar BN, McKee M, Jones L, Veizis A. Europe’s migrant containment policies threaten the response to covid-19. British Medical Journal Publishing Group; 2020. doi: 10.1136/bmj.m1213 32217531

[6] Kabir M, Afzal MS, Khan A, Ahmed H. COVID-19 pandemic and economic cost; impact on forcibly displaced people. Travel medicine and infectious disease. 2020;35:101661. doi: 10.1016/j.tmaid.2020.101661 32272198 PMC7136875

[7] Malteser International OoMwr. Keeping refugee camps free from the coronavirus pandemic 2022 [cited 2022 11/12/2022]. Available from: https://www.malteser-international.org/en/current-issues/refugees-and-displacement/coronavirus-in-refugee-camps.html.

[8] Zard M, Lau LS, Bowser DM, Fouad FM, Lucumí DI, Samari G, et al. Leave no one behind: ensuring access to COVID-19 vaccines for refugee and displaced populations. Nature Medicine. 2021;27(5):747–9. doi: 10.1038/s41591-021-01328-3 33875889 PMC10413720

[9] Doocy S, Lyles E, Akhu-Zaheya L, Burton A, Burnham G. Health service access and utilization among Syrian refugees in Jordan. Int J Equity Health. 2016;15(1):108. doi: 10.1186/s12939-016-0399-4 27418336 PMC4946096

[10] Benos A, Kondilis E, Pantoularis I, Makridou E, Rotulo A, Seretis S. Critical Assessment of Preparedness and Policy Responses to SARS-CoV2 Pandemic: International and Greek Experience. CEHP-Centre for Research and Education in Public Health, Health Policy and …; 2020.

[11] International M. Keeping refugee camps free from the coronavirus pandemic 2020 [Available from: https://www.malteser-international.org/en/current-issues/refugees-and-displacement/coronavirus-in-refugee-camps.html#:~:text=Millions%20of%20refugees%20and%20displaced,camps%20would%20have%20devastating%20consequences.

[12] Martuscelli PN. How are forcibly displaced people affected by the COVID-19 pandemic outbreak? Evidence from Brazil. American Behavioral Scientist. 2021;65(10):1342–64. 10.1177/00027642211000402 PMC7992097 38603086

[13] Abbas Kigozi CG. Access to COVID-19 vaccines for refugees in Uganda 2022 [cited 2022 11/12/2022]. Available from: https://www.oxfam.org/en/research/access-covid-19-vaccines-refugees-uganda.

[14] Control CfD. Prevention Interim guidance on management of coronavirus disease 2019 (COVID-19) in correctional and detention facilities. 2021.

[15] Malchrzak W, Babicki M, Pokorna-Kałwak D, Doniec Z, Mastalerz-Migas A. COVID-19 Vaccination and Ukrainian Refugees in Poland during Russian–Ukrainian War—Narrative Review. Vaccines. 2022;10(6):955. doi: 10.3390/vaccines10060955 35746562 PMC9230022

[16] Alio M, Alrihawi S, Milner J, Noor A, Wazefadost N, Zigashane P. By refugees, for refugees: Refugee leadership during COVID-19, and beyond. International Journal of Refugee Law. 2020;32(2):370–3.

[17] DUALE M. “To be a refugee, it’s like to be without your arms, legs”: A Narrative Inquiry into Refugee Participation in Kakuma Refugee Camp and Nairobi, Kenya. 2020.

[18] Betts A, Pincock K, Easton-Calabria E. Refugees as providers of protection and assistance. RSC Research in Brief, https://bitly/31txeP5 (Accessed 16 October 2020). 2018.

[19] UNHCR TURA. Thousands flee into Uganda following clashes in DR Congo. 2022.

[20] WHO Coronavirus (COVID-19) Dashboard [Internet]. 2023. Available from: https://covid19.who.int/table.

[21] UNHCR TURA. Uganda Refugee Settlements: COVID-19 update 2022 [Available from: https://data.unhcr.org/es/dataviz/153.

[22] Zeng K, Santhya S, Soong A, Malhotra N, Pushparajah D, Thoon KC, et al. Serial intervals and incubation periods of SARS-CoV-2 Omicron and Delta variants, Singapore. Emerging infectious diseases. 2023;29(4):814. doi: 10.3201/eid2904.220854 36878009 PMC10045676

[23] Deeks J. When can odds ratios mislead?: Odds ratios should be used only in case-control studies and logistic regression analyses. BMJ: British Medical Journal. 1998;317(7166):1155. doi: 10.1136/bmj.317.7166.1155a 9784470 PMC1114127

[24] Islam MS, Rahman KM, Sun Y, Qureshi MO, Abdi I, Chughtai AA, et al. Current knowledge of COVID-19 and infection prevention and control strategies in healthcare settings: A global analysis. Infection Control & Hospital Epidemiology. 2020;41(10):1196–206. doi: 10.1017/ice.2020.237 32408911 PMC7253768

[25] Migisha R, Morukileng J, Biribawa C, Kadobera D, Kisambu J, Bulage L, et al. Investigation of a COVID-19 outbreak at a regional prison, Northern Uganda, September 2020. The Pan African Medical Journal. 2022;43. 10.11604/pamj.2022.43.10.33598 PMC9557806 36284891

[26] Ahmed F, Ahmed Ne, Pissarides C, Stiglitz J. Why inequality could spread COVID-19. The Lancet Public Health. 2020;5(5):e240. doi: 10.1016/S2468-2667(20)30085-2 32247329 PMC7270465

[27] Abaluck J, Kwong LH, Styczynski A, Haque A, Kabir MA, Bates-Jefferys E, et al. Impact of community masking on COVID-19: A cluster-randomized trial in Bangladesh. Science. 2022;375(6577):eabi9069. doi: 10.1126/science.abi9069 34855513 PMC9036942

[28] Karmacharya M, Kumar S, Gulenko O, Cho Y-K. Advances in facemasks during the COVID-19 pandemic era. ACS Applied Bio Materials. 2021;4(5):3891–908. doi: 10.1021/acsabm.0c01329 35006814

[29] Howard J, Huang A, Li Z, Tufekci Z, Zdimal V, van der Westhuizen H-M, et al. An evidence review of face masks against COVID-19. Proceedings of the National Academy of Sciences. 2021;118(4):e2014564118. doi: 10.1073/pnas.2014564118 33431650 PMC7848583

[30] Greenhalgh T, Schmid MB, Czypionka T, Bassler D, Gruer L. Face masks for the public during the covid-19 crisis. Bmj. 2020;369. doi: 10.1136/bmj.m1435 32273267

[31] Garcia LP. Use of facemasks to limit COVID-19 transmission. Epidemiologia e Serviços de Saúde. 2020;29. 10.5123/S1679-49742020000200021 32321003

[32] Refugees’ Challenges in Camps [Internet]. 2023. Available from: https://bonyan.ngo/refugees-challenges-in-camps/.

