# Supplementary material for: Investigation of COVID-19 outbreak at a refugee transit centre, Kisoro District, Uganda, June–July 2022
Source: PLOS Glob Public Health. 2024 Mar 6;4(3):e0002428. doi: 10.1371/journal.pgph.0002428 (PMC10917256; doi:10.1371/journal.pgph.0002428)
Supplement: S1 Checklist — (DOCX) [file pgph.0002428.s001.docx]

# STROBE Statement—checklist of items that should be included in reports of observational studies

|  | Item No. | Recommendation | Page  No. | Relevant text from manuscript |
| --- | --- | --- | --- | --- |
| **Title and abstract** | 1 | (*a*) Indicate the study’s design with a commonly used term in the title or the abstract | 1,2 | Investigation of COVID-19 Outbreak |
|  |  | (*b*) Provide in the abstract an informative and balanced summary of what was done and what was found | 2 | Under **methods** and **results** section of abstract |
| Introduction | | | |  |
| Background/rationale | 2 | Explain the scientific background and rationale for the investigation being reported | 3,4 | In April 2022, the NTC registered its first case of COVID-19 through mandatory screening at entry and exit. By August 30, 2022, together with the Bubukwanga Transit Center in Bundibugyo District, the two registered a total of 1,365 COVID-19 cases |
| Objectives | 3 | State specific objectives, including any prespecified hypotheses | 4 | We investigated the outbreak to establish its scope, identify factors associated with COVID-19 infection in NTC, and to recommend control and preventive measures for the future |
| Methods | | | |  |
| Study design | 4 | Present key elements of study design early in the paper | 5,6,7,8 |  |
| Setting | 5 | Describe the setting, locations, and relevant dates, including periods of recruitment, exposure, follow-up, and data collection | 5,6,7,8 |  |
| Participants | 6 | (*a*) *Cohort study*—Give the eligibility criteria, and the sources and methods of selection of participants. Describe methods of follow-up  ***Case-control study*—Give the eligibility criteria, and the sources and methods of case ascertainment and control selection. Give the rationale for the choice of cases and controls**  *Cross-sectional study*—Give the eligibility criteria, and the sources and methods of selection of participants | 7 | We conducted an unmatched case control study. We defined a case as a person who had a negative COVID-19 test at entry and positive COVID-19 test at exit and had stayed ≥5 days at NTC between June 26 and July 16, 2022. A control was defined as person who had a negative COVID-19 test at both entry and exit and had stayed ≥5 days at NTC between June 26 and July 16, 2022. The outcome variable was COVID-19 positive at exit (for a case) and COVID-19 negative test at exit (for a control) given a negative COVID-19 test at entry |
|  |  | (*b*) *Cohort study*—For matched studies, give matching criteria and number of exposed and unexposed  *Case-control study*—For matched studies, give matching criteria and the number of controls per case | 7 | We conducted an unmatched case-control study |
| Variables | 7 | Clearly define all outcomes, exposures, predictors, potential confounders, and effect modifiers. Give diagnostic criteria, if applicable | 8 |  |
| Data sources/ measurement | 8* | For each variable of interest, give sources of data and details of methods of assessment (measurement). Describe comparability of assessment methods if there is more than one group | N/A |  |
| Bias | 9 | Describe any efforts to address potential sources of bias | N/A |  |
| Study size | 10 | Explain how the study size was arrived at | N/A |  |

Continued on next page

| Quantitative variables | 11 | Explain how quantitative variables were handled in the analyses. If applicable, describe which groupings were chosen and why |  |  |
| --- | --- | --- | --- | --- |
| Statistical methods | 12 | (*a*) Describe all statistical methods, including those used to control for confounding | 8 | Variables that had a p-value <0.2 at bivariate analysis were included in the final model for multivariate analysis in a backward stepwise approach. Corresponding adjusted odds ratios (aORs) and 95% confidence intervals were reported. The final level of significance was considered at a p-value <0.05. |
|  |  | (*b*) Describe any methods used to examine subgroups and interactions | N/A |  |
|  |  | (*c*) Explain how missing data were addressed | N/A |  |
|  |  | (*d*) *Cohort study*—If applicable, explain how loss to follow-up was addressed  *Case-control study*—If applicable, explain how matching of cases and controls was addressed  *Cross-sectional study*—If applicable, describe analytical methods taking account of sampling strategy | N/A |  |
|  |  | (*e*) Describe any sensitivity analyses | N/A |  |
| Results | | | | |
| Participants | 13* | (a) Report numbers of individuals at each stage of study—eg numbers potentially eligible, examined for eligibility, confirmed eligible, included in the study, completing follow-up, and analysed | N/A |  |
|  |  | (b) Give reasons for non-participation at each stage | N/A |  |
|  |  | (c) Consider use of a flow diagram | N/A |  |
| Descriptive data | 14* | (a) Give characteristics of study participants (eg demographic, clinical, social) and information on exposures and potential confounders | 12 | Table 1 |
|  |  | (b) Indicate number of participants with missing data for each variable of interest | N/A |  |
|  |  | (c) *Cohort study*—Summarise follow-up time (eg, average and total amount) | N/A |  |
| Outcome data | 15* | *Cohort study*—Report numbers of outcome events or summary measures over time | N/A |  |
|  |  | *Case-control study—*Report numbers in each exposure category, or summary measures of exposure | 12,13 | Table 1 and Table 2 |
|  |  | *Cross-sectional study—*Report numbers of outcome events or summary measures | N/A |  |
| Main results | 16 | (*a*) Give unadjusted estimates and, if applicable, confounder-adjusted estimates and their precision (eg, 95% confidence interval). Make clear which confounders were adjusted for and why they were included | 13 | Table 2 |
|  |  | (*b*) Report category boundaries when continuous variables were categorized | 12 | Age in table 2 |
|  |  | (*c*) If relevant, consider translating estimates of relative risk into absolute risk for a meaningful time period | N/A |  |

Continued on next page

| Other analyses | 17 | Report other analyses done—eg analyses of subgroups and interactions, and sensitivity analyses | N/A |  |
| --- | --- | --- | --- | --- |
| Discussion | | | | |
| Key results | 18 | Summarise key results with reference to study objectives | 13,14 | Having close contact with a symptomatic person increased the odds of COVID-19 infection. Overcrowding and failure to use facemasks among refugees likely fuelled the outbreak. |
| Limitations | 19 | Discuss the limitations of the study, taking into account sources of potential bias or imprecision. Discuss both the direction and magnitude of any potential bias | 15 | Firstly, refugees who met the case definition and were transferred to resettlement camps were not followed up because of logistical limitations to reach the settlements they had been settled, potentially resulting in an underestimation of the study outcomes. Secondly, incomplete records on sex, and age of refugees in the registers at the NTC may have resulted in an underestimation of disease burden. Lastly, the retrospective nature of the investigation is susceptible to recall and social desirability bias, as participants may have been inclined to provide answers that were considered appropriate. This could have resulted into an overestimation of the effect of the associated factors on the COVID-19 infection. |
| Interpretation | 20 | Give a cautious overall interpretation of results considering objectives, limitations, multiplicity of analyses, results from similar studies, and other relevant evidence | 14,15 |  |
| Generalisability | 21 | Discuss the generalisability (external validity) of the study results | N/A |  |
| Other information | |  | | |
| Funding | 22 | Give the source of funding and the role of the funders for the present study and, if applicable, for the original study on which the present article is based | 18 | The project was supported by the President’s Emergency Plan for AIDS (PEPFAR) through the United States Centers for Disease Control and Prevention Cooperative Agreement number GH001353-01 through Makerere University School of Public Health to the Uganda Public Health Fellowship Program, Ministry of Health |

*Give information separately for cases and controls in case-control studies and, if applicable, for exposed and unexposed groups in cohort and cross-sectional studies.

**Note:** An Explanation and Elaboration article discusses each checklist item and gives methodological background and published examples of transparent reporting. The STROBE checklist is best used in conjunction with this article (freely available on the Web sites of PLoS Medicine at http://www.plosmedicine.org/, Annals of Internal Medicine at http://www.annals.org/, and Epidemiology at http://www.epidem.com/). Information on the STROBE Initiative is available at www.strobe-statement.org.
