# Supplementary material for: Investigation of COVID-19 outbreak at a refugee transit centre, Kisoro District, Uganda, June–July 2022
Source: PLOS Glob Public Health. 2024 Mar 6;4(3):e0002428. doi: 10.1371/journal.pgph.0002428 (PMC10917256; doi:10.1371/journal.pgph.0002428)
Supplement: S1 Text — (DOCX) [file pgph.0002428.s002.docx]

1. **Unique study ID**
2. **Date of interview:** DD/MM/YYYY
3. **Initials of the interviewer**
4. **Tel contact:**
5. **Pick GPS coordinates** Latitude…………. Longitude …………
6. **Is respondent a:** □ National □ Refugee □ Health worker □ Other, specify: _____________________________
7. **What is the respondent’s Date of birth:** DD/MM/YYYY
8. **Respondent Sex**: □ Male □ Female
9. **Did you have a sample taken for COVID-19 (Corona) testing (swab in your nose)**? □ Yes □No 🡪 **Skip to Q12**
   1. **If YES, date(s) samples taken:** □ Sept 6 □ Sept 14/15 □ I’m not sure
   2. **If YES, was your test positive?** □ Yes □No 🡪 **skip to Q12**
10. **Date of admission to COVID 19 Isolation unit or HBC:** DD/MM/YYYY
11. **Did you ever have any of the following symptoms within two weeks of sample collection (2 weeks before and 2 weeks after)** □ Yes □No 🡪 **skip to Q12**
    1. **If YES, mark the symptoms you had:**

| □ Fever □ Chills □ Cough □ Sore throat □ Runny nose / flu□ Headache □ Chest pain □ Nausea □ Shortness of breath □ Conjunctivitis |
| --- |
| □ Vomiting □ Diarrhea □ Muscle pain □ Abdominal pain □ Joint pain |
| □ Skin rash □ Weakness □ Seizures □ Irritability/confusion |
| □ Inability to walk □ Lost consciousness □ Other, specify: ________________ |

**11. If the respondent had symptoms, date of the first symptom**: DD/MM/YYYY □ Don’t know the date

1. **Do you have special privileges that allow you to leave the camp?** □ Yes □ No 🡪 **Skip to Q17**
   1. **If YES, describe where you go when you leave the camp**: ______________________________________
   2. **If YES, how often do you leave the camp?** □ Daily □ Weekly □ Less than weekly
   3. **If YES, did you interact with other people when you were in the community?** □ Yes □ No 🡪 **Skip to Q17**
      1. **If YES, please describe**: _________________________________ □ Refused
2. **Did you have interactions with local community members around the time of COVID-19 sample collection (such as selling items in the market, etc.)?** □ Yes □ No 🡪 **Skip to Q18**
   1. **If YES, describe**: _____________________________________________________________
3. **Do you have any of the following chronic medical conditions? (Tick all that applies)**

| □ HIV □ TB □ Asthma □ Heart disease □ High blood pressure |
| --- |
| □ Diabetes □ Kidney disease □ Lung disease □ Liver disease □ Obesity |
| □ Neurologic disease (incl. epilepsy) □ Cancer, specify: ________ □ Other, specify:_____________ |
|  |

1. **For female respondents only, what is your current status?**

□ Pregnant (trimester □ Post-partum (<6 weeks after delivery) □ Breastfeeding □ Not pregnant

1. **Do you smoke?** □ Yes □ No
2. **How often do you wear a face mask (do not read to respondent)?**
   - Not at all
   - Sometimes
   - Most / all of the time
3. **Tell me all the occasions when you wash your hands in a day (do not read to respondent)?**
   - Never or almost never
   - After using the toilet
   - Before eating
   - After I touch a surface or other person
   - Other, specify: __________________
4. **Were you vaccinated against COVID-19?** □ Yes □ No

**a) If yes, last date of vaccination:** DD/MM/YYYY

**b) If yes, completion status**  □ Completed □ only first dose □ Booster dose
